# Modulation of Neurological Deficits and Expression of Glutamate Receptors during Experimental Autoimmune Encephalomyelitis after Treatment with Selected Antagonists of Glutamate Receptors

**DOI:** 10.1155/2013/186068

**Published:** 2013-07-08

**Authors:** Grzegorz Sulkowski, Beata Dąbrowska-Bouta, Lidia Strużyńska

**Affiliations:** Laboratory of Pathoneurochemistry, Department of Neurochemistry, Mossakowski Medical Research Centre, Polish Academy of Sciences, 5 Pawińskiego Street, 01-106 Warsaw, Poland

## Abstract

The aim of our investigation was to characterize the role of group I mGluRs and NMDA receptors in pathomechanisms of experimental autoimmune encephalomyelitis (EAE), the rodent model of MS. We tested the effects of LY 367385 (S-2-methyl-4-carboxyphenylglycine, a competitive antagonist of mGluR1), MPEP (2-methyl-6-(phenylethynyl)-pyridine, an antagonist of mGluR5), and the uncompetitive NMDA receptor antagonists amantadine and memantine on modulation of neurological deficits observed in rats with EAE. The neurological symptoms of EAE started at 10-11 days post-injection (d.p.i.) and peaked after 12-13 d.p.i. The protein levels of mGluRs and NMDA did not increase in early phases of EAE (4 d.p.i.), but starting from 8 d.p.i. to 25 d.p.i., we observed a significant elevation of mGluR1 and mGluR5 protein expression by about 20% and NMDA protein expression by about 10% over the control at 25 d.p.i. The changes in protein levels were accompanied by changes in mRNA expression of group I mGluRs and NMDARs. During the late disease phase (20–25 d.p.i.), the mRNA expression levels reached 300% of control values. In contrast, treatment with individual receptor antagonists resulted in a reduction of mRNA levels relative to untreated animals.

## 1. Introduction

Experimental autoimmune encephalomyelitis (EAE) is an animal model used in investigations of the pathomechanisms of multiple sclerosis (MS). MS is an inflammatory demyelinating disease of the central nervous system (CNS) that often affects young adults. The disease is characterized by damage and loss of the oligodendrocytes that myelinate axons and facilitate neurotransmission. The etiology of MS has not been established.

Recent studies have suggested that glutamate neurotoxicity may be involved in the pathogenesis of MS [1, 2]. Disturbances in glutamate levels in cerebrospinal fluid and changes in expression of ionotropic and metabotropic glutamate receptors have been observed in the brains of MS patients [3].

Glutamate is the main excitatory neurotransmitter in mammalian brain and plays an important role in both physiological and pathological mechanisms operating in the CNS. The extracellular level of glutamate must be tightly controlled because an excess of this neurotransmitter leads to overstimulation of glutamate receptors and subsequent cell death. This phenomenon is known as excitotoxicity [2, 4]. There is evidence that overactivation of glutamate receptors contributes to the process of cell death in numerous chronic neurodegenerative disorders such as motor neuron disease (MND), amyotrophic lateral sclerosis (ALS), Huntington's disease, Parkinson's disease, and Alzheimer's disease [4–9]. Oligodendrocytes, the myelin-producing cells of the CNS, are highly vulnerable to glutamate excitotoxicity. It was suggested that glutamate released by macrophages might be involved in axonal damage and oligodendrocyte pathology in MS lesions [10]. The observation that excitotoxicity is one of the pathomechanisms operating during the course of neurodegenerative diseases such as MS suggests that anti-glutamatergic agents may exert neuroprotective action.

While ionotropic NMDA-, AMPA-, and kainate-type glutamate receptors (iGluRs) mediate fast synaptic transmission, metabotropic glutamate receptors (mGluRs) (mGluR1 and mGluR5) modulate neuronal excitability and development, synaptic plasticity, transmitter release, and memory function via a variety of intracellular second messenger systems [11–13]. Functional interactions between ionotropic and group I metabotropic glutamate receptors have been identified. Electrophysiological experiments have shown functional interplay between mGlu1/5 and NMDA receptors in various structures of the brain where activation of mGlu5 receptors enhances NMDA-evoked responses [14].

Previous studies have indicated a role of NMDA receptors in the pathogenesis of EAE and in the loss of blood-brain barrier (BBB) integrity which is involved in the pathomechanisms of the disease [15]. It has been found that memantine, an antagonist of NMDARs, modifies the neurological course of EAE and prevents the breakdown of the BBB [15, 16]. In addition to NMDA receptors, group I mGluRs may participate in glutamate-mediated neurotoxicity which has been demonstrated in cultured cerebellar granule neurons [17]. Administration of the mGluR1 antagonist LY 367386 was found to induce dose-dependent partial neuroprotection. When LY 367386 was administered with MK-801, an uncompetitive NMDA receptor antagonist, complete prevention of glutamate-induced cell death was observed. Differences in the neuroprotective effects of antagonists of group I mGluRs between two ischemia models (adult gerbil model of transient forebrain ischemia versus rat model of perinatal asphyxia) were also observed [18].

We thus decided to characterize the role of mGluR G I receptors and NMDA receptors in pathogenesis of EAE and investigate the possibility of using antagonists of these receptors to modulate their expression in Lewis rats with EAE. We tested the effect of the glutamate receptor antagonists amantadine and memantine (uncompetitive NMDA receptor antagonists) as well as antagonists of mGluR G I : LY 367385 (a competitive antagonist of mGluR1), and MPEP (a noncompetitive antagonist of mGluR5) on the development of neurological symptoms during EAE. The drugs were administered individually or, with the assumption that the neuroprotective effect would be enhanced, the NMDAR antagonists were administered in combination with antagonists of group I mGluRs. We also investigated the effect of the antagonists on mRNA and protein levels of mGluR1, mGluR5 and NMDA receptors.

## 2. Materials and Methods

### 2.1. Animal Model

Procedures for all animal experiments were approved by the local Ethics Committee. Experiments were performed on female Lewis rats weighing between about 180–200 g. The rats were arranged into 7 groups (1 control group and 6 experimental groups which were subjected to different recovery periods following drug treatments). To induce experimental autoimmune encephalomyelitis (EAE), we immunized the rats subcutaneously in both hind feet with an inoculum containing guinea pig spinal cord homogenate emulsified in Freund's complete adjuvant containing 5.5 mg/mL *Mycobacterium tuberculosis* H37Ra (Difco, Detroit, MI, USA).

Rats were housed under environmentally controlled conditions and were permitted free access to food and water. Body weights and neurological deficits were determined daily according to the following scale: 0, no signs; 1, flaccid tail; 2, impairment of fighting reflex and/or loss muscle tone in hind limbs; 3, complete paralysis of hind limbs; 4, paraplegia; and 5, moribund state/death [19–21]. Sham-immunized rats (control group) received subcutaneous injections of  Freund's complete adjuvant containing *M. tuberculosis* only (Difco, Detroit, MI, USA).

Amantadine was administered at a dose of 100 mg/kg b.w./day. Memantine was administered at a dose of 60 mg/kg b.w./day. LY 367385 and MPEP were administered at doses of 10 mg/kg b.w./day. The drugs were dissolved in PBS and administered intraperitoneally to the EAE rats either separately or in combination daily for 7 consecutive days, starting from 5 d.p.i. to 11 d.p.i. 

### 2.2. Materials

During the experiments, the rats were monitored until days 4, 8, 12, 20, or 25 after the initial injection inducing EAE or after drug administration between 5–11 d.p.i. At the respective time points, four rats of each group were killed to obtain tissue for immunoblotting and real-time PCR analyses together with respective controls. The brains were rapidly removed and tissues were then frozen in liquid nitrogen and stored at −70°C for further experiments. To obtain homogenates for immunoblots, the forebrains were homogenized in 50 mM phosphate buffer (pH  7.4) containing 10 mM EGTA, 10 mM EDTA, 0.1 mM PMSF, and 100 mM NaCl in the presence of protease inhibitor cocktail (1 *μ*g/mL leupeptin, 0.1 *μ*g/mL pepstatin, and 1 *μ*g/mL aprotinin).

### 2.3. Western Blot Analysis

 Brain homogenates were subjected to SDS-polyacrylamide gel electrophoresis and examined to determine protein expression levels of NMDARs and mGluRs G I (group I of metabotropic glutamate receptors); mGluR1 and mGluR5. The protein concentration in brain homogenates was determined using the method of Lowry et al. [22]. Proteins (20 g) were separated on 10% polyacrylamide gel and transferred to nitrocellulose membrane according to the Laemmli procedure [23]. Blots were blocked in PBS buffer containing 0.1% Tween-20 and 5% non-fat milk (TPBS) for 1.5 h. After washing (3 × 10 min) in TPBS buffer, the blots were incubated overnight with primary monoclonal antibodies against NMDAR (1 : 500) or mGluRs (1 : 1000) and subsequently, after washing with TPBS (3 × 10 min), with secondary antibodies conjugated with HRP (1 : 6000). A monoclonal antibody against *β*-actin (1 : 500) was used as an internal standard. Bands were detected with the chemiluminescence ECL kit (Amersham), exposed 10–20 min to Hyperfilm ECL (Amersham), and densitometric analysis of band patterns was performed using UltraScan XL (Pharmacia).

### 2.4. Determination of the mRNA Levels of GluRs by Real-Time PCR

Total RNA was extracted from brain cortex using TRI Reagent (Sigma, St. Louis, MO, USA), and 2 g of RNA was reverse-transcribed using random primers and AMV reverse transcriptase (Applied Biosystems, Forest City, CA, USA). The RT-PCR conditions were reverse transcription at 42°C for 45 min and denaturation at 94°C for 30 s. For quantitative real-time PCR analysis, TaqMan technology was applied. The rat glutamate receptor-specific primers used are as follows: for mGluR 1-ID: Rn00566625_m1*, gene symbol Grm1; for mGluR 5-ID: Rn00566628_m1*, gene symbol Grm5; and for NMDARs ID: Rn01530724_m1*, gene symbol Narg2. The probe was obtained from Applied Biosystems (Forest City, CA, USA). In order to normalize the mRNA expression of glutamate receptors, actin levels were determined using the predeveloped TaqMan assay reagents (Applied Biosystems, Forest City, CA, USA). Real-time PCR was conducted on an ABI Prism 7500 system, using 5 *μ*L of RT product, TaqMan PCR Master Mix, primers and TaqMan probe in a total volume of 20 *μ*L. The PCR cycle conditions were as follows: initial denaturation at 95°C for 10 min, 50 cycles of  95°C for 15 s, and 60°C for 1 min. Each sample was analyzed in triplicate. The relative expression levels of the glutamate receptors were calculated using the standard curve method and normalized to actin.

### 2.5. Statistical Analysis

The results are expressed as percentages of control, and data are the mean SD from 3-4 experiments. Significance was assessed by one-way ANOVA. Dunnett's multiple comparison test was used to identify the changes that were significantly different from control values (*P < 0.05, **P < 0.01, ***P < 0.001 versus control-healthy rats) or untreated rats with EAE at corresponding d.p.i. (^#^ *P* < 0.05, ^##^ *P* < 0.01, ^###^ *P* < 0.001 versus EAE animals after therapy with antagonists).

## 3. Results

### 3.1. The Influence of Drug Administration on the Course of the Disease

**Figure 1:**
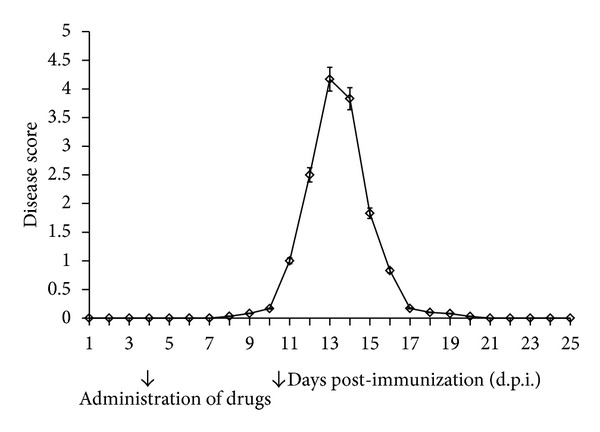
Scores of the neurological symptoms of animals during the course of  EAE. The results are means ± SD from more than 120 animals. The arrows in the diagram indicate the time point of drug administration in treated groups.

The neurological deficits observed during the course of  EAE were classified daily according to the scale from 1+ to 5+ as described in Section 2. Neurological symptoms of  EAE include progressive developmental paralysis of tail and hind limbs and reduction of physical activity in experimental rats. The neurological symptoms of  EAE started at 10-11 d.p.i. and peaked at 12-13 d.p.i. On 14 d.p.i. rats had attained partial recovery from neurological symptoms and full recovery was observed at 17 d.p.i. We did not observe any further neurological symptoms of the disease through to the end of the experiments at 25 d.p.i. (Figure 1).

We also noted changes in body weight during the course of EAE. In all experimental groups, the rats reached their highest body weight at approximately 8 d.p.i. At this time, body weights were in the same range in the EAE and drug-treated groups. Starting from 8 d.p.i. to 14 d.p.i, rats in all groups underwent a progressive 20–30% weight loss (data not shown). Detailed observations of EAE animals and clinical parameters during the experiment are presented in Table 1.

**Figure 2:**
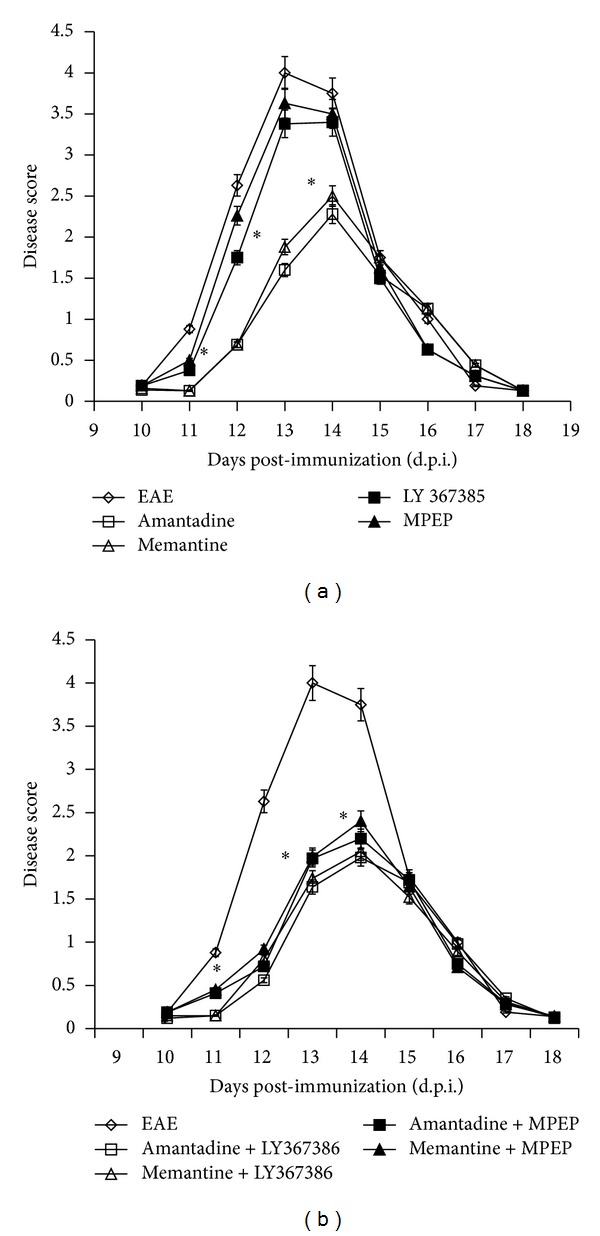
Scores of the neurological symptoms during the acute phase of EAE and after treatment with antagonists of glutamate receptors. Antagonist doses were as follows: amantadine 100 mg/kg b.w./day, memantine 60 mg/kg b.w./day, LY 367385 10 mg/kg b.w./day, and MPEP 10 mg/kg b.w./day. These doses were administered separately (a) or in combination (b) from 5 to 11 d.p.i. Neurological signs were recorded until recovery of the control EAE group at 25 d.p.i. The values indicate neurological score ± SD. Results are combined data from four to eight animals in each group. *P < 0.05; **P < 0.01 compared with untreated EAE rats.

The effects of administration of glutamate receptor antagonists (amantadine, memantine, LY 367385, and MPEP) on neurological deficits during the course of  EAE are illustrated in Figures 2(a) and 2(b) and presented in Table 1. We observed a statistically significant reduction of neurological symptoms in rats after administration of amantadine or memantine. In these experimental groups, the maximal neurological score was 2+ (flaccid tail, impairment of fighting reflex and/or loss muscle tone in hind limbs) and all experimental rats were in better neurological condition than the untreated rats with EAE. Administration of amantadine or memantine both had the effect of reducing the severity and duration of neurological deficits; the average cumulative index, duration of illness, and maximal score were reduced by factors of 8.5, 2.8, and 1.9, respectively, relative to the untreated EAE rats (Table 1). Further, the inductive phase of the disease was extended by a factor of  2.1 with administration of amantadine and memantine. We did not observe neuroprotective effects of mGluR G I antagonists. Administration of LY 367385 or MPEP did not influence neurological deficits and the condition of the experimental rats during the course of the disease while given separately (Figure 2(a), Table 1) or in combination with the NMDAR antagonists (amantadine and memantine). The neurological deficits and condition of examined animals were the same as in the case of treatment with amantadine or memantine exclusively (Figure 2(b)).

### 3.2. Changes in the Expression Levels of Glutamate Receptors after Drug Administration

To investigate the changes in protein and mRNA expression of both group I mGluRs and NMDA receptors during the course of  EAE and after therapy with glutamate receptor antagonists, we performed Western blots and real-time PCR analysis. Western blots were used to evaluate the changes in the immunocontent of receptor protein in brain homogenates obtained from control rats, rats with EAE, and drug-treated rats. A strongly positive immunoreaction was observed in a single band near 206 kDa for mGluRs G I and 180 kDa for NMDA receptors.

**Figure 3:**
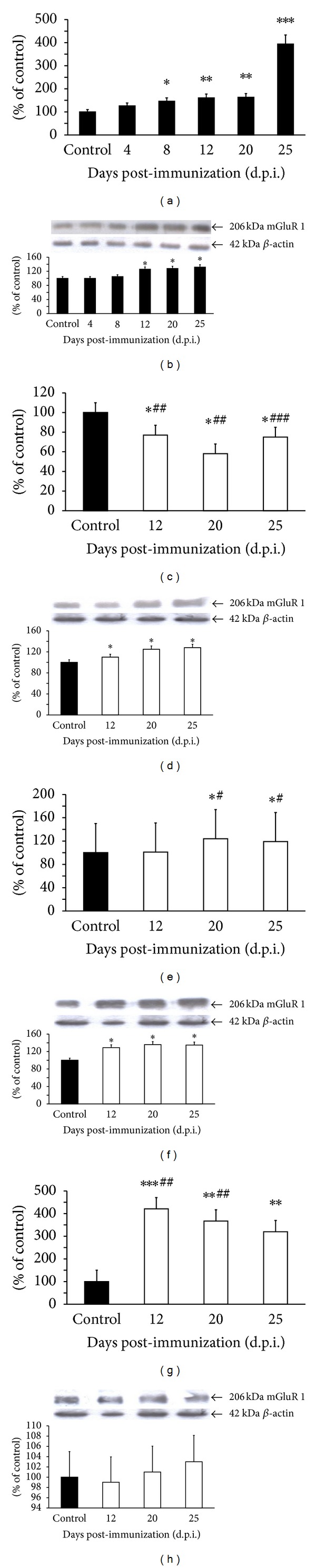
Expression of mGluR1 mRNA (a, c, e, and g) and protein (b, d, f, and h) in forebrain of control and EAE rats at different times post-immunization (a and b) and after therapeutic treatment with antagonists of glutamate receptors: amantadine (c and d), memantine (e and f), and LY 367385 (g and h). Total RNA was prepared from healthy control rats, rats with EAE, and rats with EAE after therapy at the indicated d.p.i. The mGluR1 mRNA levels were determined by quantitative real-time PCR (see Section 2) and normalized against actin. Graphs (a), (c), (e), and (g) present the results expressed as percentage of control from four independent experiments. *P < 0.05; **P < 0.01; ***P < 0.001 different versus control (healthy untreated rats). ^#^ *P* < 0.05; ^##^ *P* < 0.01; ^###^ *P* < 0.001 different versus EAE rats not subjected to therapy in corresponding d.p.i. (one-way ANOVA followed by Dunnett's multiple comparison posttest). Representative immunoblots show the expression of mGluR 1 receptor protein in forebrain homogenates of (b) control rats and rats with EAE at different times post-immunization, (d) amantadine-treated EAE rats, (f) memantine-treated rats with EAE, and (h) LY 367385-treated rats with EAE. The results are expressed as percentage of control. Graphs (b), (d), (f), and (h) present the results of densitometric analysis, normalized to *β*-actin, of four independent immunoblots, each done from distinct brain; *P < 0.05 (one-way ANOVA with post hoc Dunnett's test).

Our studies revealed changes in the level of mGluR 1 mRNA in immunized rats. Starting from 8 d.p.i. we observed a statistically significant increase in mGluR 1 mRNA, reaching 300% of the control value at 25 d.p.i. (Figure 3(a)). Changes in the mRNA level corresponded to the increase in protein expression observed from 12 to 25 d.p.i. These changes were only about 25% higher relative to the control level (Figure 3(b)). After administration of amantadine (Figure 3(c)) or memantine (Figure 3(d)), the animals developing EAE were found to have lower mGluR1 mRNA levels (by about 20% compared with control and almost 300% relative to untreated EAE rats at an appropriate time point after immunization). The expression of mGluR1 receptor protein remained at an elevated level 20%–30% above control values at 20–25 d.p.i. (Figures 3(d) and 3(f)). Administration of LY 367385 (mGluR1 antagonist) resulted in increased expression of mGluR1 mRNA by about 300% relative to control values (Figure 3(g)). These changes were accompanied by increased expression of the protein reaching 15–20% of control values (Figure 3(h)).

**Figure 4:**
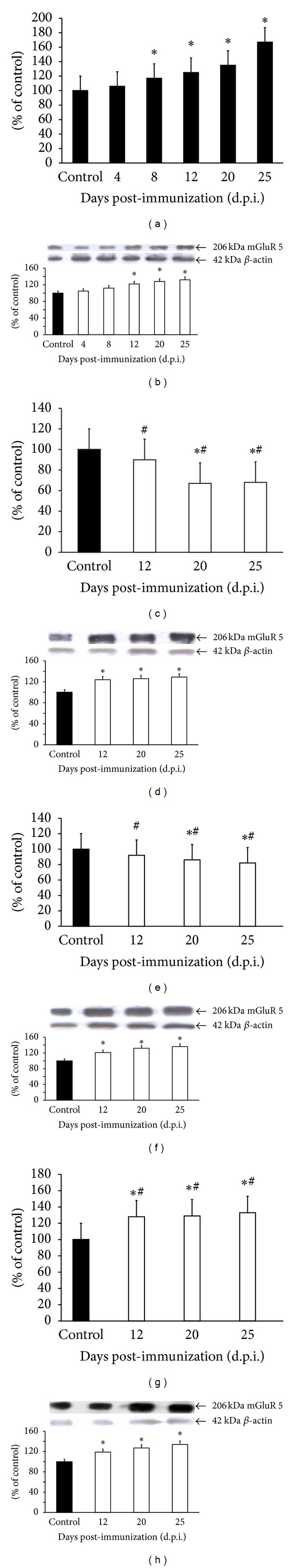
Expression of mGluR5 mRNA (a, c, e, and g) and protein (b, d, f, and h) in forebrain of control rats and rats with EAE at different times post-immunization (a and b) and after therapeutic treatment with antagonists of glutamate receptors: amantadine (c and d), memantine (e and f), and MPEP (g and h). Total RNA was prepared from healthy control rats, rats with EAE, and rats with EAE after therapy at the indicated d.p.i. The mGluR5 mRNA levels were determined by quantitative real-time PCR (see Section 2) and normalized to actin. Graphs (a), (c), (e), and (g) present the results expressed as percentage of control from four independent experiments. *P < 0.05; **P < 0.01; ***P < 0.001 significantly different versus control (healthy untreated rats). ^#^ *P* < 0.05; ^##^ *P* < 0.01; ^###^ *P* < 0.001 significantly different versus rats with EAE not subjected to therapy at the corresponding d.p.i. (one-way ANOVA followed by Dunnett's multiple comparison posttest). Representative immunoblots show the expression of mGluR5 receptor protein in forebrain homogenates of (b) control rats and rats with EAE at different times post-immunization, (d) amantadine-treated rats with EAE, (f) memantine-treated rats with EAE, and (h) MPEP-treated rats with EAE. The results are expressed as percentage of control. Graphs (b), (d), (f), and (h) present the results of densitometric analysis, normalized to *β*-actin, of four independent immunoblots, each done from distinct brain; *P < 0.05 (one-way ANOVA with post hoc Dunnett's test).

 Trends in the level of expression of mGluR5 were found to be similar to the changes in mGluR1 expression. Levels of mGluR5 mRNA increased between 12–25 d.p.i. (Figure 4(a)) by about 80% compared to controls. Elevated expression of mGluR5 protein (approximately 20%) in the same time range (12–25 d.p.i.) was also observed (Figure 4(b)). Administration of amantadine or memantine had the effect of decreasing the level of mGluR5 mRNA expression by about 20% compared to controls (normal rats) and by almost 100% compared to untreated rats with EAE at an appropriate time point after immunization (Figures 4(c) and 4(e)). In contrast, expression of mGluR5 protein was practically unchanged (Figures 4(d) and 4(f)). Administration of the mGluR5 antagonist MPEP caused an increase in the level of mGluR5 mRNA by approximately 40% over the control values (Figure 4(g)). At the protein level, mGluR5 expression was about 20% higher (Figure 4(h)).

**Figure 5:**
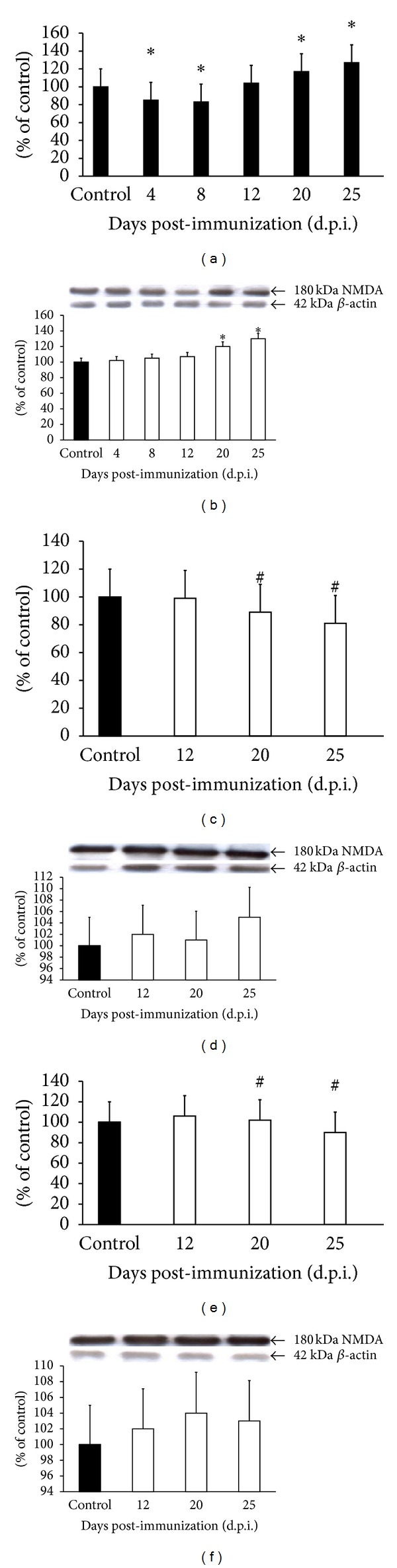
Expression of mRNA of NMDARs (a, c, and e) and protein (b, d, and f) in forebrain of control and EAE rats at different times post-immunization (a and b) and after therapeutic treatment with antagonists of NMDA receptors: amantadine (c and d) and memantine (e and f). Total RNA was prepared from healthy control rats, rats with EAE, and rats with EAE after therapy at the indicated d.p.i. Levels of NMDA mRNAs were determined by quantitative real-time PCR (see Section 2) and normalized to actin. Graphs (a), (c), and (e) present the results expressed as percentage of control from four independent experiments. *P < 0.05 versus control (one-way ANOVA followed by Dunnett's multiple comparison posttest). Representative immunoblots show the expression of NMDA receptor protein in forebrain homogenates of (b) control rats and rats with EAE at different times post-immunization, (d) amantadine-treated rats with EAE, and (f) memantine-treated rats with EAE. The results are expressed as percentage of control. Graphs (b), (d), and (f) present the results of densitometric analysis of four independent immunoblots, normalized to *β*-actin, each done from distinct brain. *P < 0.05 (one-way ANOVA with post hoc Dunnett's test).

Observations of NMDAR expression at the mRNA level revealed a reduction of about 20–30% at the early time points after immunization (4–8 d.p.i.) and a 10–15% increase (Figure 5(a)) in the later phase of the disease (12–25 d.p.i.). Enhancement of NMDA receptor protein expression by approximately 10% was observed only in the late phase (20–25 d.p.i.) (Figure 5(b)). Administration of tested NMDA receptor antagonists (amantadine and memantine) resulted in a slight decrease of NMDA mRNA (Figures 5(c) and 5(e)), while the changes in protein levels, although noticeable, were found to be statistically insignificant (Figures 5(d) and 5(f)).

 Coadministration of NMDAR antagonists (amantadine and memantine) with antagonists of group I mGluR (LY36785 and MPEP) did not have a further influence on the levels of protein or mRNA expression of the tested receptors (data not shown). 

## 4. Discussion

Glutamate is the primary excitatory amino acid in the mammalian CNS. When released from presynaptic terminals, glutamate activates the ionotropic NMDA, AMPA, and KA and metabotropic receptors (mGluRs). This can lead to the excitatory signaling which underlies processes operating during development, plasticity, learning, and memory [1, 11, 13, 24]. Glutamate is not metabolized by extracellular enzymes. Ninety percent of extracellular glutamate concentration released from nerve endings is removed by reuptake to the astrocytes and neurons. Excitatory amino acid transporters (EAATs) are involved in this process. Regulation within the synaptic cleft is critical to limit the overstimulation of excitatory amino acid receptors. Multiple neurodegenerative diseases, including MS, have been associated with changes in expression and function of glutamate receptors and glutamate transporters [1, 3, 4, 8].

Our earlier study showed changes in protein expression of glutamate transporters (GLT-1 and GLAST) in cerebellum and forebrain of rats subjected to EAE. We observed a statistically significant reduction in protein levels of both glutamate transporters in the acute phase of EAE and during the recovery (25 d.p.i.) that may result in lowering of glutamate clearance and lead to insufficient protection against glutamate excitotoxicity [25]. When the level of glutamate increases in the synaptic cleft, disturbances can occur in the signaling process and in the activity of glutamate receptors. Excitotoxic damage of nerve tissue is a common pathological event which accompanies overstimulation of glutamate receptors and changes in glutamate transport [26, 27].

In the present study, we investigated whether drugs such as LY 367385 (a mGluR1 selective antagonist) MPEP (a mGluR5 antagonist) and amantadine and memantine (antagonists of NMDA glutamate receptors, which inhibit excitatory glutamatergic neurotransmission through different mechanisms), have neuroprotective effects in the established rat model of MS. We also expected that combined treatment with NMDARs and mGluRs antagonists would furthermore improve the condition of rats with EAE. The method of administration and optimal therapeutic doses of amantadine, memantine, LY 367385, and MPEP were selected on the basis of previously published data [5, 15, 17, 28–30].

It has been generally accepted that acute excitotoxic degeneration of neurons evoked by glutamate is mediated mainly by NMDA receptors, whose activation leads to a massive accumulation of Ca^2+^ of extracellular origin inside the cells [4, 31] and further to increases in the intracellular concentrations of Ca^2+^ to pathological levels. The potential use of antagonists of  NMDARs as neuroprotective agents has been established in preclinical studies [16, 32–34]. Memantine has been found to reduce the lethality of neurons when used against NMDA- or homocysteine-induced excitotoxicity in organotypic hippocampal slices and in cultured neurons [35, 36], or against excitotoxic brain damage in animal models of diseases [15, 18]. Amantadine and memantine have also been found to be effective in relieving symptoms of multiple sclerosis or EAE pathology [28, 34, 37, 38].

Similarly, in our experiments, the antagonists of NMDA receptors were found to effectively reduce the development and duration of neurological deficits during therapy of EAE rats, and were found to be effective in modifying all of the assessed parameters of the disease. We observed reduction in neurological scores by amantadine and memantine administered prophylactically from day 7 to 11 after immunization, that is, when the symptoms of EAE were not evident. The clinical status of treated animals was significantly improved, and the severity of neurological deficits was reduced. After therapy, the disease score decreased to 2.5, while in untreated animals it remained at 4.5. In addition, the duration of disease was reduced by about 2-3 days, whereas the inductive phase was prolonged by about 2 days relative to untreated rats.

On the other hand, electrophysiological experiments have identified a modulatory effect of postsynaptically located group I mGluRs on NMDAR activity resulting in enhancement of NMDAR-evoked responses in many parts of the brain [11, 13, 39, 40]. In turn, presynaptically-located mGluRs of group I have been shown to act as release-enhancing autoreceptors mediating the acceleration of glutamate exocytosis on glutamatergic synapses [41–44]. 

Therefore, antagonists of group I mGluRs are substances which can inhibit the stimulating functions of the CNS. This appears promising for therapy of neurodegenerative disorders [45, 46]. These antagonists were also found to exhibit neuroprotection in mixed cortical neuron cultures, brain injury during ischemia and in animal models of neurodegenerative diseases [5, 15, 29, 30]. 

Based on these data, we expected to observe enhancement of neuroprotection in rats with EAE which were subjected to combined therapy with antagonists of  both types of glutamate receptors (NMDARs and mGluRs). However, our results suggest that inhibition of the NMDARs by amantadine and memantine is sufficient to ameliorate the symptoms of EAE. Antagonists of group I mGluRs (LY 367385 and MPEP) did not influence the condition of the treated animals when they were administered alone. Moreover, even when administered in combination with amantadine and memantine, these antagonists did not improve neurological deficits.

The results of our experiments indicate an increase of protein and mRNA levels of group I mGluRs (mGluR1 and mGluR5) during the course of EAE in untreated rats when compared with controls. The increased expression of mGluR1 and mGluR5 protein and mRNA levels, which started within 8–12 d.p.i. and lasted until to the end of experiment, was found be to correlated with the acute symptomatic phase of the disease. Somewhat lesser and later occurring changes in the expression of ionotropic NMDARs were observed (20–25 d.p.i.). This suggests that both types of glutamatergic receptors are involved in the pathomechanisms of EAE and may participate in excitotoxic brain damage during the course of the disease. Overexpression of glutaminergic receptors might be observed when the concentration of extracellular glutamate in the brain is increased, particularly for a prolonged period [1, 2, 7, 30]. Thus, the increased expression we identified may reflect a response to the excess of glutamate and overactivation of the glutamatergic system. Therapy with both antagonists of NMDARs (amantadine and memantine) and mGluRs (LY 367385 and MPEP) did not have a significant influence on the protein levels of the receptors. However, we observed a statistically significant difference in mRNA expression of the receptors in the treated animals. Administration of both amantadine and memantine was found to decrease expression of mGluR1 and mGluR5 mRNA gradually to the lowest level at 25 d.p.i. The duration of the experiment was probably sufficient to evoke the response in expression of mRNA but not in expression of protein.

Blocking of NMDA receptors with amantadine and memantine induces feedback action to decrease the levels of glutamate released from synaptic vesicles into extracellular space, which, in the longer timeframe of 20–25 d.p.i., results in reduced expression of mRNA for both mGluR1 and mGluR5. The opposite effect was found to be induced by mGluR antagonists (LY 367385 and MPEP). This suggests that, despite blocking of mGluRs by their antagonists, the excessive release of glutamate to the extracellular space is not inhibited and receptors remain over-stimulated. Indeed, it has been shown that blocking of NMDARs decreases the feedback of synaptically released glutamate [9, 12, 26, 47]. This shows that NMDA receptors play an important role during the course of EAE. 

Thus, it appears that exclusively blocking the activity of NMDARs, rather than total blockade of different types of glutamate receptors, is sufficient to obtain effective reduction of the consequences of elevated glutamate levels in rats with EAE. This finding is in agreement with the results of a previous study of mice with EAE in which prophylactic administration of riluzole, an inhibitor of glutamate-dependent neurotransmission, was found to reduce neurological severity, inflammation, demyelination, and overstimulation of GluRs [48]. 

## 5. Conclusions

The results of our study demonstrate that some glutamate receptors are implicated in neurodegenerative processes which occur during EAE pathology. Data from the literature indicate various mechanisms of functional coupling between group I mGluRs and NMDARs. This indicates that excitoxicity and neurodegenerative processes occurring during the course of EAE may be mediated by the cooperative action of group I mGluRs and NMDARs. Indeed, the antagonists of NMDARs (amantadine and memantine) exert the neuroprotective effect and significantly inhibit the neurological deficits in rats with EAE. However, a neuroprotective effect was not observed when group I mGluRs antagonists (LY 367385 and MPEP) were administered separately or in combination with amantadine or memantine. Neither the general condition of the experimental animals nor the neurological deficits were improved. However, all of the antagonists investigated in this work have the effect of modifying the expression of mRNA but not protein expression of group I mGluRs and NMDARs relative to the untreated rats with EAE.

## Figures and Tables

**Table 1:** Characterization of the EAE animal model and clinical parameters in rats with EAE and after treatment with antagonists of glutamate receptors.

	EAE	Amantadine	Memantine	LY 367385	MPEP
Animals with clinical sings (%)	96.2	100	100	100	100
Animals with severe EAE (%)	73.9	62.7*	63.6*	73.6	70.0
Lethality (%)	8.8	0	8.3	0	0
Inductive phase (days)	10.6 ± 2.4	12.1 ± 1.3*	12.2 ± 2.1*	10.8 ± 1.4	10.5 ± 1.5
Maximal CI (score)	4.5 ± 0.3	2.4 ± 0.4*	2.6 ± 0.6*	4.1 ± 0.6	3.9 ± 0.5
Cumulative CI (score)	28.6 ± 4.7	19.6 ± 1.6*	20.1 ± 2.4*	26.98 ± 0.5	27.6 ± 0.7
Duration of disease (days)	21.4 ± 1.8	18.6 ± 1.6*	17.8 ± 2.1*	20.98 ± 1.9	20.2 ± 1.2
Number of animals	160	24	24	24	24

The values represent the means ± SD. *P < 0.05, significantly different when compared with rats with EAE.

## Abbreviations

AMPA: *α*-Amino-3-hydroxy-5-methyl-4-isoxazolepropionate
ANOVA: Analysis of variance
d.p.i.: Days post-immunization
EAATs: Excitatory amino acid transporters
EAE: Experimental autoimmune encephalomyelitis
GLAST: Glutamate-aspartate transporter
GLT-1: Glutamate transporter 1
GluRs: Glutamate receptors
iGluRs: Ionotropic glutamate receptors
KA: Kainate type glutamate receptors
LY 367385: (S-2-Methyl-4-carboxyphenylglycine)-competitive antagonist of mGluR1
mGluRs: Metabotropic glutamate receptors
MPEP: (2-Methyl-6-(phenylethynyl)-pyridine)-the mGluR5 antagonist
MS: Multiple sclerosis
NMDA: N-Methyl-D-aspartate
NMDARs: NMDA receptors.
